# Using Chromatin-Nuclear Receptor Interactions to Quantitate Endocrine, Paracrine, and Autocrine Signaling

**DOI:** 10.1177/1550762919899643

**Published:** 2020-02-27

**Authors:** Matthew D. Taves, Jonathan D. Ashwell

**Affiliations:** 1 National Institutes of Health, Bethesda, MD, USA

**Keywords:** autocrine, endocrine, glucocorticoids, glucocorticoid receptor, paracrine, steroid transport, steroids, steroidogenesis, transcription factor

## Abstract

Hormone-activated nuclear receptors (NRs) control myriad cellular processes. The classical paradigm for hormone delivery is secretion from endocrine organs and blood-borne distribution to responding cells. However, many hormones can also be synthesized in the same tissues in which responding cells are found (paracrine signaling). In both endocrine and paracrine signaling, numerous factors affect hormone availability to target cell NRs, including hormone access to and sequestration by carrier proteins, transport across cell membranes, metabolism, and receptor availability. These factors can differ dramatically during development, between anatomical locations, and across cell types, and may cause highly variable responses to the same hormone signal. This has been difficult to study because current approaches are unable to quantify cell-intrinsic exposure to NR hormone ligands, precluding assessment of cell-specific hormone access and signaling. We have used the ligand-dependent interaction of the endogenous glucocorticoid (GC) receptor with chromatin as a biosensor that quantifies systemic access of GCs to cells within tissues at the single cell level, showing that tissues are buffered against circulating GCs. This approach also showed highly targeted paracrine GC signaling within the thymus, where GCs promote the positive selection of thymocytes with moderate affinity for self-antigens and the development of a safe and effective T-cell repertoire. We believe that this and complementary biosensor approaches will be useful to identify endocrine and paracrine target cells in situ and quantify their exposure to hormones regardless of the mode of delivery.

**Comment on:** Taves MD, Mittelstadt PR, Presman DM, Hager GL, Ashwell JD. Single-Cell Resolution and Quantitation of Targeted Glucocorticoid Delivery in the Thymus. Cell Rep. 2019 Mar 26;26(13):3629-3642.e4. doi: 10.1016/j.celrep.2019.02.108. PubMed PMID: 30917317; PubMed Central PMCID: PMC6447081.

## Introduction

Nuclear receptors (NRs) are a group of structurally related proteins that function as ligand-dependent transcription factors and play critical roles in cell development, differentiation, activity, and death. Nuclear receptors bind and are activated by small hydrophobic ligands that include steroids, oxysterols, vitamin D, retinoids, thyroid hormones, and bile acids (the ligands for certain “orphan” receptors are unknown). Liganded NRs undergo a conformation change that releases chaperone proteins and exposes a DNA-binding domain containing highly conserved zinc fingers, which allows DNA binding and regulation of gene expression. Because of their wide-ranging and pleiotropic functions, synthetic NR ligands are among the most commonly prescribed treatments for metabolic, inflammatory, degenerative, autoimmune, and neoplastic diseases.^1^

## Endocrine Signaling

The canonical model of hormone action involves endocrine signaling: secretion from one organ and blood-borne delivery to distant targets. This is the primary pathway by which many NR ligands (eg, steroids, bile acids, vitamins) are understood to signal. Here, we will focus on a well-studied example: adrenally produced glucocorticoids (GCs) that bind the ubiquitously expressed glucocorticoid receptor (GR). Stress-induced increases in GC secretion induce a complex systemic response including gluconeogenesis in the liver, increased cardiovascular output, enhanced cognition, suppressed reproductive functions, and suppressed immunity.^2^ In this way, GCs function as bona fide global signals orchestrating an adaptive stress response.

Closer examination, however, shows that GC responses are highly cell-specific, with the quality and degree of responsiveness varying greatly between even closely related cell types.^3^ The ability of different cell types to mount unique responses to similar GC exposure is a consequence of multiple variables that influence the liganded GR, including posttranslational modifications, available dimerization partners and coregulators, and chromatin accessibility.^4^ Another variable, often overlooked, is GC access to the GR. It is generally taken for granted that circulating GCs have free and unfettered access to the GR, and that GRs of cells throughout the body are exposed to similar GC levels. This follows from the idea that GCs (and other NR ligands) are thought to passively exit endocrine organs, travel through the bloodstream, diffuse into tissues throughout the body, and bind their receptor. However, in reality, this is unlikely to be the case, as significant hurdles exist at each of these stages. Being lipophilic molecules, GCs bind to hydrophilic carrier proteins such as corticosteroid-binding globulin (CBG) and albumin to travel through the blood. Thus, exit from a producing cell and transfer to such carriers present an initial barrier. Similarly, binding to carrier proteins buffers circulating GCs and only a fraction (~5%) is free at any time to cross the vascular endothelium and enter target cells.^5^ Of this free fraction, selective entry into and export from cells can be regulated by membrane channels,^6^ and importers may even be required for entry of some steroids.^7^ Finally, within the target cell, enzymes can modify GCs to either increase or decrease receptor binding.^8^ Thus, the bioactive steroid concentration to which the receptor in any given cell in a tissue is exposed is determined by multiple prereceptor biophysical and biochemical factors. Determining the aggregate result of these factors requires determining the ligand level actually sensed by the receptor itself.

**Figure 1.**
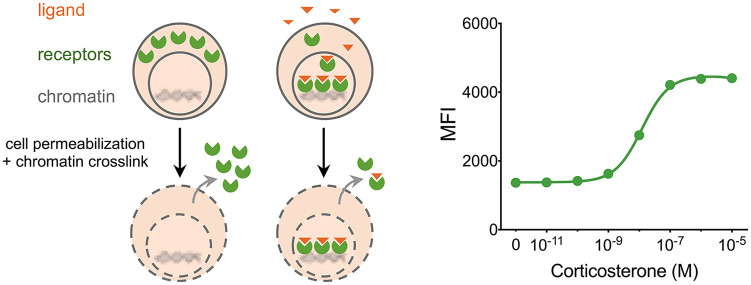
Permeabilization fixation retains ligand-bound nuclear receptors in the cell. *Note.* Treatment of cells with FACS buffer (PBS [phosphate-buffered saline] with 2% FCS [fetal calf serum], 0.5% BSA [bovine serum albumin], 0.05% sodium azide) containing detergent (0.5% Triton X-100) and fixative (2% formaldehyde) preferentially retains nuclear receptors in the cell while depleting unliganded receptors (left). A representative dose-response graph (right, adapted from Taves et al^10^) demonstrates retention of a GFP-GR fusion protein after treatment of cells with different concentrations of corticosterone for 30 minutes. Data were acquired by flow cytometry, and median GFP intensity is shown for each dose. GR = glucocorticoid receptor.

Established techniques allow extraction and measurement of total steroid levels in blood and solid tissues, and while these are often similar, they can differ dramatically in certain tissues and at different developmental timepoints.^9^ Physical removal of carrier-bound GCs allows the measurement of free (unbound) steroid levels available to circulating cells.^5^ Notably, the levels of bioactive GCs within tissues are unknown. To bypass this limitation, we recently developed an approach that allows use of the endogenous GR itself as a faithful detector of bioavailable GCs.^10^ Because only the liganded GR associates with chromatin, we used simultaneous detergent permeabilization and formaldehyde crosslinking to wash out unliganded receptors and selectively retain liganded and chromatin-crosslinked GRs (Figure 1). Fluorescently labeled antibody staining of cells after this “perm-fix” treatment and subsequent analysis by flow cytometry revealed that the retained GR signal correlated well with GC levels over a physiological range of concentrations. The same pattern held for other NRs, such as androgen, estrogen, and progesterone receptors, demonstrates the generalizability of the approach to the study of other ligand-dependent NRs.

**Figure 2.**
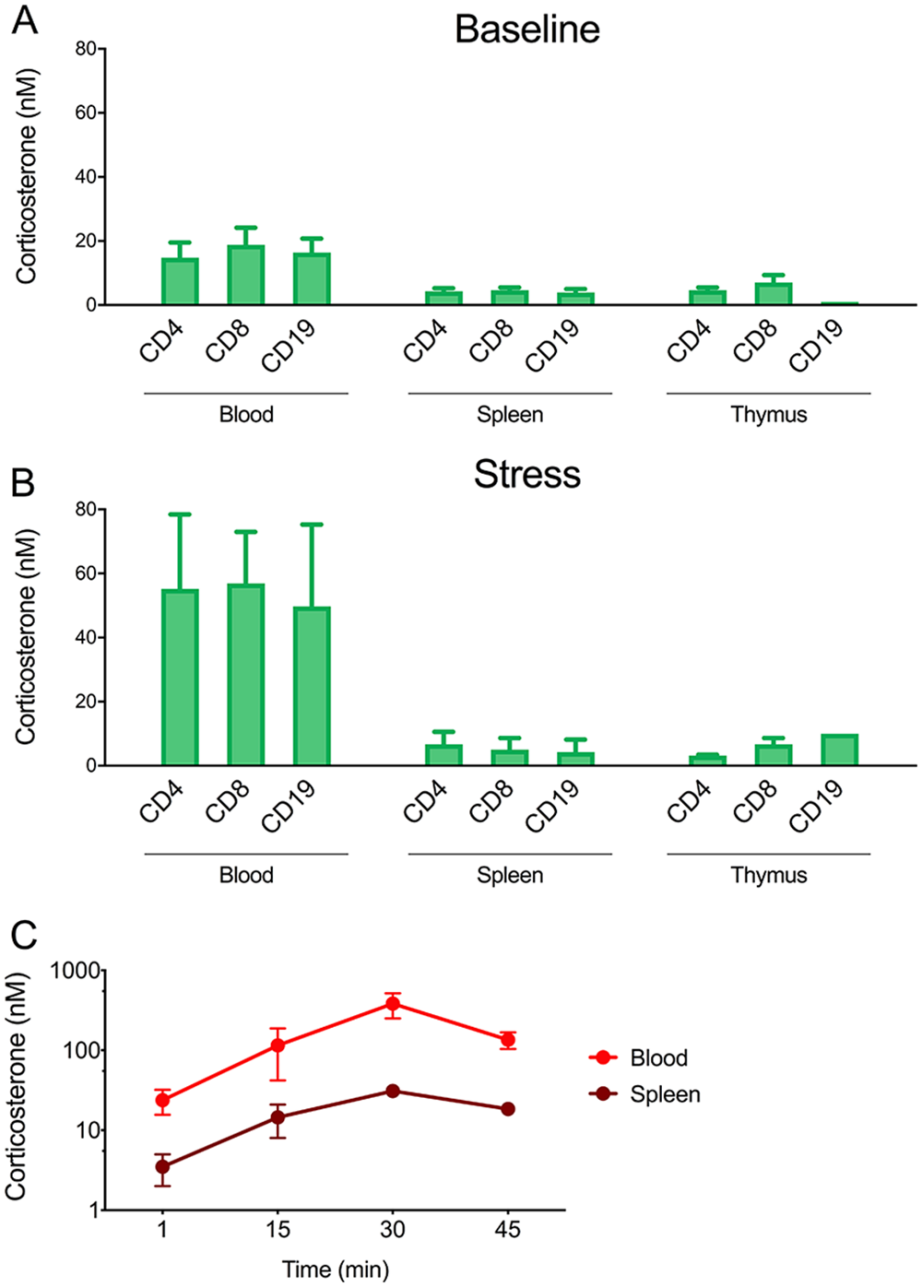
Bioactive GC concentrations of lymphocytes in mouse blood and lymphoid organs. *Note.* GC exposure of CD4^+^ T cells, CD8^+^ T cells, and CD19^+^ B cells in the blood, spleen, and thymus of GFP-GR mice (A) at baseline (euthanized within 2 minutes of initial disturbance; adapted from Taves et al^10^) or (B) after 15 minutes of mild stress. (C) Kinetics of bioactive GC levels in the blood and spleen were determined by collecting samples after various durations of mild stress. In all experiments, mice were rapidly euthanized, blood and tissue cells collected, and cell aliquots treated with a range of corticosterone concentrations. Cells were analyzed by the permeabilization fixation and ligand titration assay as described.^10^ GC = glucocorticoid; GR = glucocorticoid receptor; GFP = green fluorescent protein.

**Figure 3.**
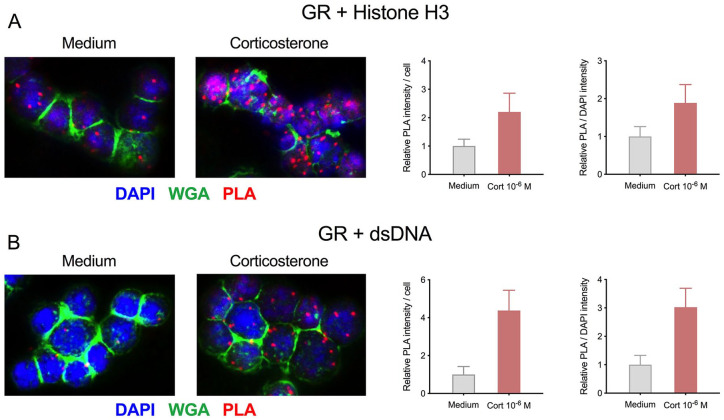
Detection of GC-induced GR-chromatin interactions using PLA. *Note.* Thymocytes were incubated in medium with or without 1 µM corticosterone for 30 minutes, fixed in 4% paraformaldehyde followed by ice-cold methanol and PLA (Sigma Duolink Orange) used to detect closely co-colocalized (A) mouse anti-GR and rabbit anti-histone H3 or (B) rabbit anti-GR and mouse anti-double-stranded DNA following the manufacturer’s protocol. DAPI (4′,6-diamidino-2-phenylindole) and Alexa 488–conjugated wheat germ agglutinin (WGA) were used to detect nuclei and plasma membranes, respectively. Confocal images (left) were analyzed with ImageJ (≥244 cells per treatment/antibody combination; right). GC = glucocorticoid; GR = glucocorticoid receptor; PLA = proximity ligation assay.

To quantitate endogenous cell-specific GC exposures, we used a secondary ligand titration assay in which rapidly collected cells were aliquoted into multiple wells and briefly exposed to various GC concentrations before perm-fix treatment, GR staining, and flow cytometry. Added (secondary) concentrations above the endogenous (primary) exposure would increase GR retention, whereas concentrations similar to or lower than the endogenous exposure would have little effect, allowing one to estimate endogenous cell-specific bioavailable GC levels. Using this “endogenous biosensor” approach, we determined the GC levels that mouse lymphocytes perceive in vivo. In the blood of both unstressed and stressed mice, the cell-specific GC exposure of lymphocytes (CD4^+^ T cells, CD8^+^ T cells, and CD19^+^ B cells) were the same as free GC concentrations measured by immunoassay, providing proof of principle for the assay.^10^ Interestingly, using this approach, we have found that circulating lymphocytes of unstressed mice are exposed to GC levels of ~18 nM, whereas the same cells in the spleen or thymus are exposed to only ~5 nM, approximately 28% of the blood level (Figure 2A). This blood-tissue difference remained, or was even increased, after a mild 15-minute stressor: lymphocytes in blood were exposed to GC levels of ~54 nM, whereas those in spleen or thymus remained at ~6 nM, 11% of the circulating bioavailable level (Figure 2B). These data suggest that GCs, rather than diffusing freely into tissues and cells, have regulated access. It is unlikely that the difference between blood and tissue is simply due to delayed delivery because the stress-induced increase in GC signaling follows identical kinetics in the blood and the spleen (ie, the kinetics of rise and fall are identical (Figure 3C). Decreased bioavailability in tissues may thus reflect barriers to entry and/or metabolism. The ability to measure in-organ GC bioavailability makes it now possible to quantitatively assess the relative contributions of various factors (physical diffusion, aqueous barriers and transport proteins, endothelial exclusion, target cell export, and metabolism) that could contribute to such a buffering effect in vivo. It also makes it possible to directly assay the kinetics and bioactivity of different endogenous and synthetic GR ligands.^1,6,9,11^ Finally, it allows detection of cell-specific variability in GC exposure, which could result from differences in anatomic context or expression of genes that alter GC access and activity.

## Paracrine and Autocrine Signaling

Although NR signaling is primarily thought to be a response to blood-borne hormone delivery, it has become clear that organs and tissues also regulate hormone signaling autonomously in a highly orchestrated fashion. As mentioned above, GC signaling in tissues and cells may be dramatically altered via hormone sequestration, transportation, and metabolism, and these processes are likely to vary from organ to organ, across regions within organs, and between different cell types. In addition, GCs may themselves be synthesized within target tissues, and there is increasing recognition that paracrine signaling, in which ligand produced by one cell signals a nearby receptor-expressing cell, and autocrine signaling, in which ligand is produced by and signals in the same cell, are superimposed on systemic fluctuations. For example, GC can by synthesized at extra-adrenal sites including thymic epithelial cells (TECs),^12,13^ intestinal epithelial cells,^14,15^ and skin melanocytes.^16^ In each of these cases, GC synthesis appears to inhibit immune activation and function as a local immunoregulatory loop, allowing local GC activity while avoiding the need for systemic elevation that might have untoward effects on cells and tissues in other locations.^17^ Such identification of local production raises several questions: (1) What cells are signaled by paracrine GCs? (2) Do paracrine GCs signal regionally and nonspecifically, or target specific cells in the vicinity? and (3) What are the concentrations to which local target cells are exposed?

In the mouse thymus, TEC-derived GCs ensure production of a competent repertoire of mature T cells and effective adaptive immunity.^12,18^ Thymocytes express a random array of T-cell antigen receptors (TCRs) with varying affinity for self-antigens presented by major histocompatibility complex molecules, and during their development undergo a selection process that removes cells with high affinity (negative selection) and allows the survival of cells bearing TCRs with useful affinity (positive selection). In the absence or even partial loss of thymocyte GC signaling, the affinity threshold between positive and negative selection is reduced, and thymocytes that would normally survive instead die.^18^ To identify paracrine targets of thymic-derived GC, we applied the perm-fix approach and found that TEC-produced GCs, rather than signaling cells throughout the organ, affected thymocytes at the antigen-signaled CD4^+^8^+^ (double positive) TCR^hi^ stage, a population that constitutes only a few percent of the total thymocyte pool.^10^ The ligand titration assay provided the first quantitative estimate of paracrine GC signaling: targeted cells were exposed to concentrations approximately 3-fold higher than other thymocytes. This is a satisfying observation because these thymocytes are in direct contact with TECs and activated by TEC-presented self-antigens, and because even a 50% reduction in thymocyte GR signaling is sufficient to weaken the TRC repertoire.^18^ Thus, although TECs are few in number and the GC they secrete are undetectable at the whole-organ level, TEC-proximal thymocytes with antigen-reactive TCRs are exposed to elevated GC concentrations that promote survival and positive selection. Such targeted delivery may in part result from such close contact between TEC and thymocyte: apposed cell membranes at and around the TEC: thymocyte immunological synapse may avoid buffering by CBG and albumin and result in much more efficient GC delivery. Thymocyte GC signaling may be further promoted by 11β-hydroxysteroid dehydrogenase type 1 (Hsd11b1), which regenerates active GC from inactive metabolites, amplifying intracellular GC concentrations.^11^ We hypothesize that GC synthesis, degradation/regeneration, and transport combine with anatomical localization to promote targeted paracrine signaling in the thymus.

## Receptors as Endogenous Biosensors

As outlined above, pairing chromatin-crosslinking with fluorescently tagged fusion proteins or antibody staining and flow cytometry has allowed us to begin exploring cell-specific exposure to endocrine and paracrine GC signals. As this approach worked as well with the androgen receptor, estrogen receptor, and progesterone receptor, we believe that this could be adapted for other NRs and possibly also other transcription factors whose access to chromatin is actively regulated (eg, by ligands, phosphorylation, inhibitors). In particular, we believe this chromatin-crosslinking approach is well suited to examine (1) bioavailable ligand concentrations and how they are regulated, (2) heterogeneous intra-organ ligand exposure, and (3) novel or unknown ligands, as no a priori knowledge of the ligand(s) is needed.

Although relatively straightforward, this approach has some important limitations. First, the technique is time-sensitive. On removal of ligands, the GR and other NRs dissociate from chromatin and the signal obtained by chromatin-crosslinking quickly decreases. GR-chromatin interactions were found to have a half-life of ~15 minutes after GC removal. This is slow enough to afford good temporal specificity for cells that can be quickly isolated and analyzed by perm-fix, such as the blood and lymphoid organs, but is not as suitable for organs such as the brain or gut, which require prolonged digestion to dissociate cells. Second, the use of rapid cell dispersal results in the loss of spatial information (location in the organ). Thus, while suitable for specific identification of target cells, the anatomical location of these cells within an intact organ cannot be simultaneously obtained. Anatomic location must instead be obtained by parallel experiments using approaches such as immunofluorescence microscopy or histocytometry. Third, unless one has introduced a fluorescently tagged fusion protein, the detection of chromatin-crosslinked receptors requires antibody staining, which may be costly or even impossible if there is no suitable antibody. Finally, by its very nature, chemical crosslinking kills the cells of interest, making it impossible to follow the dynamics of NR signaling in living cells. Thus, experiments investigating the kinetics and regulation of such signaling must use different samples for different timepoints or treatments, which increases noise and makes detection of subtle differences much more difficult. Improvements or adaptations of this general approach might, however, circumvent these limitations. To study paracrine and autocrine signaling, chromatin crosslinking of cultured tissue slices could allow the identification of signaled cells within a partially intact anatomic structure. Organoids might prove especially amenable to such an approach, mimicking endogenous cellular interaction and structure, but in samples small enough that permeabilization-fixation may still work to selectively retain chromatin-associated receptors.

## Conclusions

The use of receptor-chromatin binding may be broadly applicable as a measurement of ligand availability, and combining this with other experiment, techniques could help answer many outstanding questions in NR biology. One such technique is the proximity ligation assay, in which a probe is used to detect 2 primary antibodies from different species when in close proximity. Using this approach, we have been able to detect increased association between receptor and chromatin by pairing antibodies against GR with antibodies against histone H3 or double-stranded DNA (Figure 3). Such an approach allows detection of receptor-chromatin association in any fixed sample while retaining intracellular proteins of interest and complete anatomical context. More sophisticated extensions of such a biosensor approach, however, will be especially useful for detection in live cells. This might be achieved using genetically encoded biosensors, following design principles such as that used for the GCaMP family of calcium biosensors.^19^ Luciferase-based NR-based sensors have been used to detect ligands in vitro and in vivo,^20^ and the generation of fluorescent analogs would open the door to detection of cell-specific signaling dynamics in living cells and tissues. Together, we believe that NR biosensors will prove to be useful in understanding endocrine, paracrine, and autocrine signaling and be especially useful for understanding how different cells respond to these signals to regulate diverse aspects of physiology.
